# Implementation of birth plans integrated into childbirth preparation classes for vaginal birth: a qualitative study of women, their husbands and clinicians’ perspectives in Iran

**DOI:** 10.1186/s12884-022-05305-7

**Published:** 2022-12-27

**Authors:** Zaynab Mohaghegh, Mojgan Javadnoori, Mahin Najafian, Simin Montazeri, Parvin Abedi, Ehsan Kazemnejad Leyli, Shahla Bakhtiari

**Affiliations:** 1 Midwifery Department, School of Nursing and Midwifery, Ahvaz Jundishapur University of Medical Sciences, Ahvaz, Iran; 2 Reproductive Health Promotion Research Center, Department of Midwifery, School of Nursing and Midwifery, Ahvaz Jundishapur University of Medical Sciences, Ahvaz, Iran; 3 Department of Obstetrics and Gynecology, School of Medicine, Fertility, Infertility and Perinatology Research Center, Ahvaz Jundishapur University of Medical Sciences, Ahvaz, Iran; 4 Department of Midwifery, Menopause Andropause Research Centre, Ahvaz Jundishapur University of Medical Sciences, Ahvaz, Iran; 5 Biostatistics Department, Guilan Road Trauma Research Center, Guilan University of Medical Sciences, Rasht, Iran; 6 Midwifery Department, Rosie Hospital, Cambridge University Hospitals NHS, Cambridge, UK

**Keywords:** Birth plan, Experience, Qualitative research, Vaginal birth

## Abstract

**Background:**

Understanding women’s experience of birth planning is necessary for introducing and implementing this process in the Iranian maternity services. This study aims to explore perceptions of birth plan implementation in Iran from the perspective of women, their husbands, and clinicians.

**Methods:**

This qualitative study was conducted in Iran. Qualitative data were collected from November 2020 to March 2021 by conducting semi-structured in-depth interviews with ten mothers who prepared a birth plan, and 15 key informants (obstetricians, midwives, and husbands) who were involved in the implementation process of birth plans. Data were analyzed using conventional qualitative content analysis.

**Results:**

Data reduction process resulted in 380 codes that were categorized in 16 subcategories and five main categories. The main categories were “Guide and pattern of preparing for childbirth pathway”, “Maternal empowerment and sense of triumph”, “Facilitating and enhancing communication”, “Successful transition to parenthood and women’s satisfaction”, and “Challenges associated with implementation of the birth plan”. The overarching theme “Birth plan: The missing link in promotion of vaginal birth in Iran” was constructed from these categories.

**Conclusion:**

Findings of this study highlight the effectiveness of the implementation of birth plan along with childbirth preparation classes for increasing the likelihood of a successful vaginal birth and promoting empowerment and satisfaction in women during the childbirth process. The findings of this study could pave the way for developing, introducing, and implementing of birth plan in Iran.

**Supplementary Information:**

The online version contains supplementary material available at 10.1186/s12884-022-05305-7.

## Background

The childbirth process is a sacred event which creates life-long memories [1]. It affects the well-being of the mother and baby and creates bonds between them, and the new family [2]. This period is accompanied with positive experiences such the woman’s feeling of empowerment in her role as a mother and strengthened emotions during her transition to motherhood [3]. However, it may also be associated with negative maternal experiences which may significantly increase the risk of adverse health outcomes for the mother, including postpartum psychological disorders with potential long-term effects on the mother, the baby, and the whole family [4].

Every mother would like her childbirth process to be replete with beautiful and memorable moments. To achieve this goal, careful planning is required for and by women so as to experience the birth process safely and comfortably [1]. Childbirth is a wonderful yet intense process of transformation from a pregnant woman to a mother. However, inadequate knowledge of women and their fear of unknown events during pregnancy and childbirth can results in anxiety and worry for women [5]. Understanding the birth process and what is expected to happen before and after childbirth can minimize childbirth trauma [6]. In addition, planning the birth during the antenatal period improves health education and facilitates communication between women and health professionals [7].

A birth plan is a written document in which pregnant women can describe their expectations and preferences regarding the care they would like to receive during labor and childbirth [8]. Women using birth plans can reflect their personal values, needs and choices with respect to their care practice and the interventions they (do not) want to receive during childbirth [9]. The birth plan focuses on woman-centered care [10]. When care focuses on an individual woman, there is the potential to create situations where she can become more powerful, and thus strengthen her family, community, and society[11].

In 1996, the World Health Organization (WHO) introduced the birth plan as one involving “good practices in the care provision during delivery and birth” in order to reorganize and humanize obstetric care [12]. Based on the principle of bioethics of autonomy, birth plans enable women to have more control over their birth process, because it serves as an important tool in preparing for childbirth, reduces women’s fears arising from inadequate information and communication, and improves their reflection on and participation in decision-making [13]. The birth plan is considered as a tool that can reduce labor interventions, facilitate communication between the mother and her health care provider, enhance women’s feeling of empowerment, and improve childbirth outcomes [14], and all of these contribute to maternal satisfaction with the experience of childbirth [13]. In spite of their remarkable benefits, however, birth plans may be rigid and unrealistic, which may lead to conflicts and negative experiences that could affect obstetric outcomes [15]. According to a recent narrative review, there are both benefits and challenges to the preparation and use of birth plans, but that many health professionals are still in favor of using birth plans [16].

Research has shown that the prevalence of negative childbirth experiences among primiparous Iranian women is 37%, which is much higher than that in other countries [17]. Unnecessary medical interventions are still very common during the childbirth process in Iran compared to developed countries [18]. For example, performing an episiotomy or using oxytocin during labor without informed consent of women is commonplace in Iran [19], and this could be considered as an obstetric violence [20]. Furthermore, the majority (75%) of Iranian women participating in one study reported they had experienced one or more types of disrespectful maternal care, and half of them reported that they did not even have the right to move during labor and adopt their birth position of choice [21].

The use of a birth plan has become increasingly popular in western countries in defense of women’s autonomy during childbirth [22]. Although the WHO recommends birth planning as part of prenatal care [23], this recommendation has not been welcomed within the Iranian childbirth context. In addition, in some studies conducted in Iran, the quality of maternity service has been reported as moderate to poor, which needs to be revised effectively in order to provide high-quality and safe woman-centered maternity care services for women and their family [24].

Given the conflicting results of previous studies with respect to the use of birth plans [15, 25, 26] and the dearth of studies exploring the perspectives of Iranian women on the effect of birth plan on their childbirth experiences, we conducted a large mix-methods study to assess the effect of birth plan implementation on maternal and neonatal outcomes. We also carried out a qualitative study with women having vaginal birth, and their care providers and husbands, to explore their perceptions about the effect of birth plan on their childbirth experiences. In this paper, we report the results of the qualitative study.

## Study methods

### Design and setting

The present study is part of mixed methods research conducted in fulfilment of a doctoral dissertation in midwifery. It aimed to explore women, their husbands and clinicians’ perspectives of birth plan implementation in Tehran, Iran. This research was situated within a feminist pragmatism theoretical framework as its research method because it was concerned with both the midwives, who have a female-dominated profession, and women’s birth choices. [27].

Feminist pragmatism is rooted in feminist theories according to which construction of knowledge is inherently a political matter [28]. However, this alignment of pragmatism is not based on feminist theories as sociological movements. Instead, it is an epistemological and philosophical movement [29]. A qualitative content analysis approach was used to explore the mother’s experience of birth plan during pregnancy and birth, from the perspectives of women themselves, their husbands and clinician informants.

### Participants

Ten women who had participated in the quantitative research phase and had a vaginal birth were selected. To further enrich the study, interviews were also conducted with 15 key informants (two obstetricians, nine midwives, and four husbands) in line with the purpose of the study. Written informed consent was obtained from all participants. To ensure that participants were well informed about the content of the consent form, at first the lead investigator briefly explained the contents of the birth plan form as well as the goals and reasons for doing the research. A purposeful sampling method was used to select the participants for interview. The participants’ demographic data are shown in Table 1.

**Table 1:** Demographic Characteristics Women (*N* = 10) and Husbands (*N* = 4)

Variable	N (%)
**Women**	
**Age group** (years)	
18–25	3(30)
26–35	7(70)
**Education level**	
High school	4(40)
University	6(60)
**Employment status**	
Housewife	7(70)
Employee	3(30)
**Gravidity**	
1	5(50)
≥ 2	5(50)
Total	10 (100)
**Husbands**	
**Age group** (years)	
18–35	2(50)
36–42	2(50)
**Education level**	
High school	0(0)
University	4(100)
Total	4(100)

### Setting

The study setting included four public health centers (Meysam, Ayat, Afarinesh and Azadegan) affiliated to Tehran University of Medical Sciences, two private hospitals, and one hospital of armed forces (Omid Hospital, Ansari Hospital, and Najmiyeh Hospital) in Tehran, Iran. The main reason for choosing these hospitals was that the birth plan is not routinely implemented in Iran, and these hospitals facilitated pregnant women to give birth in maternity wards based on their birth plan. These hospitals manage 2000, 3000, 5000 births per year, respectively. The hospitals had to meet some conditions for the implementation of birth plan. These included the presence of the husband or a companion of the woman’s choice during the birth process, having a private midwife, having a private room, having a doula, and water birth in the maternity wards. Midwives in any Iranian hospital, including the mentioned hospitals, work under the supervision of obstetricians.

### Data collection

Data were collected from November 2020 to March 2021 via in-depth semi-structured interviews with women ten days after childbirth (Table 1). In this study, in addition to mothers, the views of midwives, obstetricians, and husbands as key informants were investigated. One of the researchers (ZM, a PhD student and a highly experienced midwife) collected the data via face-to-face or telephone interviews at a time and place convenient to the participants. Due to the COVID-19 pandemic quarantine in Iran, some of the interviews were conducted by phone. A recent study reports that qualitative interviews performed through video, telephone, and online are valid and trustworthy alternatives to traditional face-to-face interviews [30]. Telephone interviews allow a greater opportunity to interview in areas that are difficult to reach for various reasons (e.g. war zones, areas with extensive spread of infectious diseases, or areas with unstable internet connection)[30].

Interviews with women included the following questions: “Would you please describe your perception of your birth plan?”, “How do you think this birth plan affected your childbirth experience?”, “How did you write your birth plan? Explain.“,“ What challenges did you have?”. Interviews questions with the other informants included following questions: “What do you think about the birth plan and what experiences did you have?”, " What challenges did you have with implementation of birth plan?”.

Exploratory and elaborative questions were asked when more clarifications were needed.

Data saturation was achieved after 20 interviews; however, data collection continued for five more interviews to ensure that no new themes emerged. Each interview lasted from 40 to 70 min. The interviews were recorded using a voice recorder after obtaining permission from the participants. During the study period, the first author answered women’s question and responded to their concerns. No woman needed external counseling or referral due to potential stress.

### Data analysis

Conventional content analysis was used to analyze the data according to Graneheim and Lundman’s in five stages [31]. Data collection and analysis were done concurrently. First, the interviews were recorded and transcribed verbatim. Secondly, the first author read and re-read each interview several times to gain an overall impression, and the meaning units were identified and discussed by the second and third authors (MJ, SM). Thirdly, codes were identified based on the condensed meaning units (the constellation of words sentences or paragraphs containing aspects related to each other through their content and context), and then they were classified into subcategories and categories (group of content that shares a commonality) based on conceptual and semantic similarities. Finally, after comparing and contrasting the categories and subcategories together, based on similarities and differences, the main theme (the means of linking the underlying meanings together in categories) was extracted. Data analysis was conducted using MAXQDA software (version 2020). Four criteria of “credibility”, “transferability”, “dependability”, and “confirmability” were taken into account for establishing the trustworthiness of our qualitative study [32]. To ensure the credibility of the study, we used “peer debriefing” and “member checks” i.e. the extracted codes and categories were reviewed by supervisors who were expert in qualitative data analysis, at first separately and then in a meeting where some controversies regarding codes and categories were discussed. Adjustments were implemented whereby some codes and categories were refined. Additionally, to avoid uncertainty regarding some the meaning of some units or codes, the codes and categories were returned to the participants to ensure that our understanding of the meaning of their statements was correct.

To enhance dependability, data analysis was confirmed by two authors who were familiar with the analysis process. To establish confirmability, the description of the research steps was transparently reported and then confirmed by all co-authors. Furthermore, characteristics of the study setting and the participants, data collection and analysis were thoroughly described in order to allow readers to judge the transferability of the findings [31, 33].

## Results

The characteristics of the mothers, their husbands, and the caregivers (midwives and obstetricians) are summarized in Tables 1 and 2, respectively. The mean age of the women was 29.5 years. After reduction and classification of primary codes and initial categories, a total of 380 codes were extracted from the interviews, which were grouped into 16 sub-categories forming 5 main categories, and one theme (Table 3). The overarching theme was “Birth plan: The missing link in promotion of vaginal childbirth in Iran”.

**Table 2:** Demographic Characteristics of Midwives and Obstetricians (*N* = 11)

Variable	N	(%)
**Specialty**		
Midwife	9	(81.8)
Obstetricians	2	(18.1)
**Degree**		
Bachelor of Midwifery	3	(27.2)
Master of Midwifery	5	(45.4)
Ph.D. of Reproductive Health	1	(9.09)
Obstetricians	2	(18.1)
**Duration of work experience**		
10 Years	3	(27.2)
≥ 10 Years	8	(72.8)
Total	11 (100)	

**Table 3:** Subcategories, categories and theme regarding participants perspectives of birth plans

Sub-categories	Main categories	Theme
1- Identifying the goal and making informed decisions 2- Enhancing the power and creativity of the mind 3-Step-by-step guide to visualizing the vaginal birth process	Guide and pattern of preparing for childbirth pathway	**Birth plan: The missing link in promotion of vaginal childbirth in Iran**
1- Increasing the women’s skills in managing labor pain 2- Acquiring the required information from various sources 3- Increasing the mothers’ self-confidence, self-esteem, and self- efficacy. 4- Mothers’ positive attitude toward the vaginal birth	Women’s empowerment and sense of triumph	
1-Building a relationship of woman- midwife mutual trust 2-Strengthening the intra-organizational relationship among the birth team 3-Facilitating and maintaining the tripartite mother-midwife-physician relationship 34-Facilitating the women’s communication with peers	Facilitating and enhancing communication	
1-Successful transition from pregnancy to parenthood 2- Husbands’ participation in planning and implementation of the birth plan 3-Choosing a midwife as the best birth attendant	Successful transition to parenthood and women’s satisfaction	
1-Inadequate attention to planning and policy- making to promote vaginal birth 2-Conflicts between midwives and obstetricians	Challenges associated with implementation of the birth plan	

### Guide and pattern of preparing for childbirth pathway

Participants identified birth plans as models and guides, which have significant positive effects on determining the path to the ultimate goal of vaginal birth. From the point of view of mothers as well as the birth attendants participating in this study, the birth plan is a guide that helps mothers to know their expectations and preferences, to predict the requirements of childbirth, and to make informed choices accordingly. This category involves three sub-categories: “Identifying the goal and making informed decisions”, “Enhancing the power and creativity of the mind”, and “Step-by-step guide to visualizing the vaginal birth process”.

### Identifying the goal and making informed decisions

One of the participants commented: “It’s very important for the mother to know what’s going to happen to her; even for someone who wants to choose a cesarean section to go and investigate about it and know the process and to know what she wants to go through when having a birth plan, and how it’s going to be done; that’s very helpful and very important in making the path easier.” (Woman 4).

According to the mothers in this study, a birth plan serves as a guide to remind them of things at the time of childbirth that they may forget.

“The birth plan was somewhat a reminder of some of the things I had forgotten, such as which hospital I wanted to go to if my baby had any problem or whether the hospital has the facilities; for example, is there a cameraman available in the hospital at the time of the baby’s birth.” (Woman 1).

### Enhancing the power and creativity of the mind

The interviewees in this study believed that the birth plan was a model that could enhance their creativity and mentality to achieve a successful vaginal birth. To them, a birth plan was a written document that is effective in boosting and increasing the brain’s energy to achieve maximum positive results from childbirth. In fact, from participant’s point of view, since the birth plan is written, this writing process becomes a factor which helps the mother to adhere to the implementation of its provisions. They believed that the efficiency of thinking is enhanced by writing and having a plan.

“You don’t know how writing does wonders. The brain releases many times more energy for doing the things that are written. When you write your plan, it is as if you’ve passed 80% of the path. Writing a birth plan for my wife brought the best results, and I would certainly say we could not have succeeded without this plan.” (Father 2).

### *Step-by-step guide to visualizing the vaginal birth process*

The participants believed that birth plan was a step-by-step guide to visualizing the birth process and specifying the direct path to reach the goal.

“Nine months of hardship is summarized in four hours. When you have a plan, it is as if you have a guide and you reach the goal through a direct and main route, which is the vaginal birth.” (Woman 6).

In their experience, the birth plan determines the steps to reach the goal and reveals a picture of the birth process to them in advance, and writing it removes the mental ambiguities about birth.

“If we push someone into a pool full of water in a dark night, they may be worried, even if they know how to swim. Birth plan is an opportunity to prepare the woman for birth in a way that she can have a vivid picture of the process.” (Midwife 2).

### *Maternal empowerment and sense of triumph*

The women participating in this study reported that the birth plan helped them to focus only on the birth process at the time of birth and to make the right decision in admission to the hospital when their labor pain started. They also believed that the birth plan made them take responsibility for their choices.

In this study, the mothers stated that they improved their self-efficacy and empowerment for childbirth through the birth plan. This category is divided into four subcategories: “Increasing the women’s skills in managing labor pain”, “Acquiring the required information from various sources”, “Increasing the mothers’ self-confidence, self-esteem, and self- efficacy and “Women’s positive attitude toward vaginal birth”.

### *Increasing the women’s skills in managing labor pain*

Women believed that the birth plan mentally prepared them for adapting to labor pain and consciously accepting it. In their experience, the birth plan helped them have the ability to endure the labor pain.

“Basically, you need to plan for everything, especially when it comes to giving birth to a baby. Because I had no knowledge about childbirth and it was my first experience, the birth plan helped me a lot. When you have the knowledge and information, you are more determined and you make solid decisions, and this helps your mind to be ready and accept the situation, and the situation will become easier for you.” (Woman 4).

“Labor pain is a strange pain, and everyone experiences it in a different way, and I can’t say that it was more than what I had imagined or less than that, but the important thing is that I was able to endure it. This endurance was achieved thanks to the grace of God and the birth plan. What really impressed me was the pre-planning and relaxation. Although I was not mentally prepared for that amount of pain, I was more conscious about it.” (Woman 6).

From the point of view of the birth attendants, a birth plan increases the mother’s acceptance skills under unexpected circumstances during vaginal birth. They also believed that women’s minds are expanded by giving them the right to choose and increase their level of knowledge, and in this way, their inner commitment and responsibility to meet their needs in the birth plan is enhanced.

“This is the first time a birth plan is used. The woman is given the right to choose from among a lot of options. You have all these options for the birth process, which you need to choose from. When the mother sees these options and when she realizes that she knows nothing about them, she goes and collects information, so her level of knowledge is enhanced. When the level of knowledge rises, the mindset becomes wider, making the mother more successful in the selection process; and because the woman herself has made a decision, she will hold herself more responsible for this decision.” (Midwife 2).

“Decision making entails responsibility. That’s why when the mother writes her birth plan, she is responsible for the choices she makes, so she is not passive in this process; this is why this way of intervention and involving the mother in her birth can be effective. (Midwife 5)

### *Acquiring the required information from various sources*

The women in this study believed that the birth plan gave them an opportunity to think, and it motivated them to seek information from a variety of authoritative sources such as childbirth preparation classes, reputable websites and books, and other mothers’ experiences, which led to an increase in their knowledge and awareness.

“We can’t write a plan without preparation; childbirth can be done by everyone, even animals. So, since we are humans, there must be a difference between us and the animals; it is wisdom and contemplation, so we must be prepared and have a precise plan for our birth. Now, everyone gets this wisdom from somewhere. I read a lot of books, and I highlighted the main points of the books, and used to tell my husband a summary of the important points.” (Woman 7).

### *Increasing the mothers’ self-confidence, self-esteem, and self-efficacy*

According to the women participating in this study, a birth plan increased their self-confidence and reduced their fear of birth. By the same token, the birth attendants believed that having a choice in the birth plan and the effort a woman makes for it would increase her self-confidence.

“My body was fully prepared for the childbirth because I had increased my knowledge about vaginal birth. It was as if I knew how to relieve my pain by myself and how I could control myself. When you are aware of something, you are less afraid of it.” (Woman 6).

“The birth plan empowers the woman because she has manipulated her environment; she has made a choice; she has made an effort. Behind this birth, the woman’s world grows; she gains self-confidence because she gets the answer to the choice and effort she has made, so she works with self-confidence.” (Midwife 3).

### *Mothers’ sense of positive attitude towards vaginal birth*

The women participating in this study felt unique and believed that the birth plan empowered them and encouraged them to have a vaginal birth with a birth plan in subsequent births.

“If I decide to get pregnant again, I will definitely go the same way and make up for the shortcomings of my previous pregnancy.” (Woman 1).

### Facilitating and enhancing communication

The majority of the women in this study believed that a birth plan would provide an opportunity to deepen reciprocal relationships of trust and respect between women, their peers, and their caregivers. This relationship is characterized with respect to the mother’s values ​​and in gaining her trust. According to the women in our study, the mother-midwife relationship is strengthened in light of midwife’s empathy and her enthusiasm in helping the mother, which is fulfilled by providing the right training and information for the mother and encouraging and informing her about her capabilities in the birth plan. This will in turn provide an opportunity for the mother to tell the obstetrician and the midwife what she wants or does not want with respect to her birth. Having people share the same objectives and by strengthening the teamwork of members of the birth team, the birth plan facilitates and enhances the tripartite mother-midwife-obstetrician relationship. The category of Facilitating and enhancing communication had the following sub-categories: “Building a relationship of woman-midwife mutual trust”, “Strengthening the intra-organizational relationship among the birth team”, “Facilitating and maintaining the tripartite mother-midwife- physician relationship”, and “Facilitating the women’s communication with peers”.

### Building a relationship of woman- midwife mutual trust

Women stated that birth plan deepened their relationship with the midwives. They highlighted that midwives encouraged them about their capabilities in their birth plan.

“After 32 weeks, I decided to refer to a midwife. I trusted her a lot. The positive energy she gave me which made me believe that I could do it, deepened our relationship.” (Woman 3).

“This birth plan was an opportunity to talk to my midwife and doctor about anything I wanted or did not want.” (Woman 5).

From the point of view of midwives, the spirit of birth plan is respect and honoring for mother.

“The first thing that one feels in the spirit of this work is the respect for the mother and honoring her. When we ask a mother about something, we are actually respecting and honoring her, which is definitely pleasing to God, and this is a very important issue in my opinion, which we see in the birth plan.” (Midwife 5).

### *Strengthening the intra-organizational relationship among birth team*

From the point of view of the birth attendants, the birth plan is a tool to transfer the mother’s preferences from the realm of health to the realm of treatment. By establishing a purposeful relationship between the mother, the care team, and the treatment team, the intra-organizational communication is enhanced.

“When a mother with a birth plan is referred to the hospital by a health center and that mother is welcomed by an already prepared team, she will definitely have peaceful mind and experience pleasant and desirable moments during her childbirth process. This will also remove stress from the treatment and health teams.” (Midwife 2).

### Facilitating and maintaining the tripartite mother-midwife-obstetrician relationship

According to both the women and the birth attendants, the birth plan provides an opportunity to quickly transfer information between the mother, the midwife, and the obstetrician.

**“**By reading the birth plan, the midwives exactly knew what I wanted and conveyed it to the obstetrician. And that was great because when you’re in labor you don’t feel like talking. In fact, they know what you want from the moment you enter the birth room.“ (Woman 2).

### *Facilitating the women’s communication with peers*

Participants in our study believed that the birth plan facilitates the mother’s communication with other peers by providing the opportunity for communication and group discussion and exchange of information related to the birth plan.

“During pregnancy, women are most motivated to get information. When mothers as a group discuss the birth plan, this creates more communication between pregnant mothers and provides an opportunity to learn through their experiences in similar situations.” (Midwife 3).

### Successful transition to parenthood and women’s satisfaction

The majority of the women in this study were of the opinion that having a birth plan was effective in creating satisfaction as well as positive and pleasant experiences from the birth and the postpartum period. In the postpartum period, they expressed a feeling of happiness, triumph and greater readiness for parental responsibility. For some women, giving birth according to their preferences was the reason for their happiness and experiencing no depression after childbirth. In addition, the majority of mothers attributed the positive experiences and success of using the birth plan to the participation of their husbands in the planning process and supporting them during childbirth, and the choice of the midwife as the birth attendant.

This category included three subcategories: “Successful transition from pregnancy to parenthood”, “Husbands’ participation in planning and implementation of the birth plan”, “Choosing a midwife as the best birth attendant”.

### *Successful transition from pregnancy to parenthood*

“I think having a birth plan is worth it. After giving birth, one seems to be more prepared to become a mother or a father, and I don’t know what’s going on. I don’t know if this is because of team work or because the father sees what the mother goes through and gets a better understanding of her. I mean the quality of the relationship between my husband and I has improved since my last birth.” (Woman 3).

“Even though I was in so much pain and it was all annoying, I did not get postpartum depression. Although I had depression during pregnancy, I did not get postpartum depression and the main reason for this success is that I was able to give birth the way I wanted. I miss the day I gave birth.” (Woman 7).

In the women’s experience, the birth plan enhanced their motivation to breastfeed the baby with love and affection.

“Even though I knew that not all my plans might be fulfilled, it was worth it and the benefits were greater. For example, the moment you breastfeed, you milk your baby with love.” (Woman 6).

### *Husbands’ participation in planning and implementation of the birth plan*

Women stated that the birth plan helped them to know that their husbands could be on their side during childbirth. From the point of view of women, the participation of their husbands in the implementation of birth plan were very useful, and improved the progress of labor.

“My husband and I both think it is important to have a birth plan. This gave me the power to choose and helped me to know that my husband could be by my side during childbirth. It was very important for me to have my husband by my side, because it was more encouraging than when I’m alone; it also affected the progress of my birth, and it also had a great impact on my mental condition.” (Woman 4).

### *Choosing a midwife as the birth attendant*

The women interviewed in this study described the pleasant experience of childbirth as a miracle, thanks to the presence of a capable midwife.

“I was lucky enough to be able to carry out my birth plan with an experienced midwife, and I really think that the delivery itself is a miracle.” (Woman 3).

### Challenges associated with implementation of the birth plan

According to the experiences of some of the participants, the birth plan was accompanied with challenges and problems during the implementation phase. Factors such as medical dominance in Iran, the existing regulations in the delivery rooms, and mothers’ lack of access to one-to-one midwifery care were among the problems and challenges of implementing the birth plan. This category includes two subcategories: “Inadequate attention to planning and policy making to promote vaginal birth”, and “Conflicts between midwives and gynecologists”.

### Inadequate attention to planning and policy making to promote vaginal birth

The participants’ statements indicate that the main problems against implementation of the birth plans roots in the higher levels of management and policy making in health system of Iran. Problems such as forced parturient to accept the conditions in the delivery room, the lack of midwives’ authority, leadership role and decision-making power in implementation of birth plans, neglecting to provide on- demand care for women in health policies.

“I believe the success of the birth plan lies in the one-to-one midwifery care, which is pretty common in the world but still needs to be expanded and implemented in Iran.” (Midwife 6).

“We don’t have full authority to decide on the birth plan, and this poses a very big challenge to the realization of this process, and the solution is to change the policies in such a way so that we (midwives) can be responsible for the birth, which is a long way to go.” (Midwife 4).

“In Iran, we have not learned to be demanding people, and the parturient has to accept the conditions in the delivery room. For example, a pregnant mother can demand that her husband be with her during the birth. But in many good hospitals, this demand is ignored because the doctor and the maternity ward staff, who are all females, want comfortable clothing, and they consider the presence of a male stranger to be somewhat intruding, although we know that the presence of the husband is an important part of physiological childbirth.” (Midwife 1).

### *Conflicts between midwives and obstetricians*

Most midwives believe that achieving the goal and success in implementation of birth plan requires a strong teamwork. Some midwives believe that recognition of midwifery profession and midwives’ competencies by gynecologists, can lead to mutual respect and cooperation. Realization of the birth plan will benefit from such a constructive atmosphere.

“Our gynecologists think that vaginal birth is exclusively their own duty. However, everywhere in the world, vaginal birth is the midwives’ responsibility, but in our country they do not accept this, and gynecologists consider themselves to be in charge of it, and as long as they interfere with our work, they affect vaginal birth, which also affects the birth plan.“(Midwife 4).

## Discussion

This qualitative study was conducted with the aim of gaining a deeper understanding of women’s views and perceptions of using birth plan during pregnancy, childbirth, and the postpartum period in Iran. The findings demonstrated that the birth plan increased women’s positive childbirth experience and reduced their fear of vaginal birth. Moreover, it helped women to strengthen the reasoning power and better understanding about the birth process, helped women to engage actively in labour, be empowered to increase their skill for self-control and self- efficacy in labor and delivery, supported them to make informed decision, and facilitated communication with gynecologists, midwives, and other peers.

In this study, using birth plan was a new experience for the participants, and they perceived it as a model and guide that affects their preparation for a vaginal birth. The birth plan helped women to better understand their expectations and preferences for childbirth and to make the best choice for controlling labor pain based on their preferences and the knowledge they had gained. In addition, the birth plan gave the women the capability to better manage their minds for birth and focus more on the birth process. The birth plan served as a model according to which the pregnant women were able to actively participate in decision-making for their childbirth through a step-by-step visualization of the childbirth process, from the onset of pain to the birth stage. Most importantly, childbirth in this study was cited to be a positive and enjoyable experience that was achieved when the demands and expectations in the birth program were met. In this study, 66% of the pregnant women were primiparous, and they often felt anxious about their pregnancy and childbirth. However, they reported that using a pre-arranged birth plan helped them clear up their mental ambiguities about birth. It was also believed to help the mothers identify their needs earlier and enter the birth process with a better mental preparation.

The results of this study are supported by previous studies on the benefits of birth plans. In a study on 68 Chinese women, for example, women who used a birth plan stated that they perceived it as a guide or reminder to plan their care during birth [34]. Capitulo et al. believe that the birth plan helps women to identify their thoughts, preferences, and needs in relation to childbirth [35]. A review by Divall et al. reported that birth plans help enhance women’s awareness of the available options and their sense of control during childbirth [36]. In Pennell et al., the majority of women participating in the study perceived the birth plan as a useful enlightening tool which improves childbirth experiences [37]. Evidence has also shown that women’s requests may be sometimes ignored under the unpredictable and unexpected labor conditions, leading to women’s dissatisfaction with their childbirth experience [38]. Therefore, it is necessary to have a flexible approach to using the birth plan due to the possible failure of the plan and the unpredictability of the nature of childbirth[36]. In addition, it is necessary to have a dynamic understanding of this tool. Under such circumstances, the pregnant woman will have the power to give birth the way she wants, and her caregiver will be responsible for managing the changes and sharing decisions, especially when interventions are initially undesirable but increasingly necessary for the safety of the mother and/or the fetus [39]. In this regard, it is essential to consider the birth plan as a “living” and “evolving” document that may be modified with the emergence of new information and the changing conditions in the birth process [40].

Women in the present study reported that the birth plan helped them acquire the skills to accept unexpected circumstances in the birth process and to be responsible for their choices. In addition, they were of the opinion that the birth program enabled them to acquire the necessary knowledge and information from reputable sources. In order to make an informed choice, they would seek help from reputable websites, books, and the successful experiences of postpartum women. In this way, they would feel empowered and triumphant. Women’s enhanced knowledge and skills, along with their increased self-confidence and sense of overcoming the fear of vaginal birth granted them self-efficacy for vaginal birth. Pregnant women’s caregivers also believed that the birth plan increased these women’s self-confidence by giving them the right to choose and participate in the decision-making process. Anderson et al. found that postpartum self-esteem scores were higher in women who had a birth plan [41]. A qualitative study in Brazil examining the impact of birth plan on women’s empowerment showed that the birth plan empowers women in the birth process because they all felt that they are the protagonists in the childbirth process, that their body physiology is respected, and that an enjoyable and unforgettable moment had been created for them [39]. However, Malacrida and Boulton take a critical view of the ideas of “choice”, “control”, and “empowerment”, which are central concepts in birth plan. They argue that such concepts can be misleading because although women may feel that they are empowered when using a birth plan, this empowerment is lost amidst the challenges of implementing the plan in a highly medicalized model of childbirth, resulting in feelings of frustration and failure in the requested plan [42].

Participants in the present study stated that the birth plan facilitates and maintains a relationship of mutual respect and trust between women, their caregivers, and their peers. This relationship was maintained throughout labor and birth because the women believed that they were sufficiently prepared for unpredictable situations, and their caregivers were confident that these women would maintain the necessary flexibility and trust in their caregivers. Our findings are consistent with the results of previous studies reporting that all women and their caregivers considered the birth plan as a valuable communication and education tool [34, 36, 37, 40]. In Anderson et al.’s study, women with a birth plan had a higher postpartum communication score [41]. However, in Thompson et al.’s study, organizational and professional constraints in supporting women’s expectations and preferences and limiting women in their choice of personal care options were reported to compromise the relationship model between women and their caregivers from pregnancy to childbirth[43, 44]. In our study, by contrast, the women were not restricted in their choice of options, and the study was performed in private hospitals that were eligible for vaginal birth.

In our study, the majority of women were satisfied with having a birth plan. They experienced positive emotions such as happiness, success, greater readiness to accept the parenthood responsibility, and the mutual comfort between them and their husbands. A number of women in this study, including mothers with a history of previous cesarean section, were satisfied with preferring a midwife to a physician as the birth attendant. By choosing a midwife in their birth plan, they experienced reduced stress during pregnancy and enhanced childbirth. Some women even wanted to share the sweet and enjoyable experience of vaginal birth and recommended it to other expectant mothers. One of the mothers, for example, expressed her happiness about her success in the planned vaginal birth and wished that these conditions would be provided for all Iranian women. She described childbirth as a miracle in her life and asked the study researchers to let the voice of Iranian women be heard by policymakers; that they want their childbirth to be vaginal. Redshaw et al. (2019) suggested that a woman’s experiences of obstetric care may affect her future pregnancies as well as decisions about her subsequent deliveries[45]. In this study, a number of women stated that they would like to experience a vaginal pregnancy and vaginal birth again. One of them said that in her next pregnancy, she would definitely use a birth plan and would not repeat the mistakes she made in this pregnancy and birth.

Other studies have reported conflicting results on the effect of birth plan on women’s satisfaction. Farahat et al., for instance, reported that the mean score of childbirth satisfaction was significantly higher in women using a birth plan [46]. In Sham et al., the majority of the participants experienced positive emotions and satisfaction [34]. However, in Mei et al., a large number of requests included in the birth plan were associated with a decrease in satisfaction with the birth experience [47]. In our study, most of the requests made by the women were related to the presence of their husbands as their companion and midwives as agents of birth. The majority of women in this study felt highly satisfied with the participation and support of their husbands in planning the birth and believed that the birth plan increased the fathers’ level of knowledge about childbirth and controlling maternal stress in cases of risk factors. One of the mothers, who had experienced childbirth with the presence and participation of her husband addressed the mothers who are planning to become pregnant in the future as follows: “Be sure to experience vaginal birth with your husband because it will be the best birth possible.” (Woman 1).

Despite the women’s satisfaction, a number of women and midwives complained about the challenges and problems they faced in implementing the birth plan. Medical dominance in Iran, where the midwife is not in charge of vaginal births and there is no one-by-one midwifery care, accounts largely for the failure of planned vaginal births. In addition, according to the midwives participating in this study, childbirth preparation classes were not fully efficient. They argued that although childbirth preparation classes were successful in giving awareness to the mother, they failed to prepare the mother for a good childbirth. This is where the birth plan can be of great help by letting the mother contemplate and write down how her birth should be carried out. A number of midwives in this study also believed that professional challenges between midwives and obstetricians in Iran along with the midwives’ lack of decision-making power could also lead to the failure of the birth plan. However, one of the obstetricians interviewed in this study believed that the cumbersome rules related to blood money (Blood money or Diya in Islamic law, is the financial compensation paid to the victim or heirs of a victim in the cases of murder, bodily harm or property damage by mistake) force doctors to do something that they themselves might not be happy to do. Other studies reported different challenges of birth plan. For example, in Whitford et al.’s qualitative study, lack of a clear understanding of the goals of the birth plan, providing no support for the selected plans, and reluctance to do too much planning were the challenges of the birth plan [48]. Aragon et al. listed unrealistic expectations, inflexibility, and a sense of false self-control as some of the challenges of using birth plans [40].

### Strengths and limitations

This study is worthwhile in that it is the first qualitative study to examine women’s perceptions of the impact of the birth plan on their childbirth experience while attending childbirth preparation classes in Iran. However, like any other study, it has a number of limitations. First, due to the COVID-19 pandemic and the lockdown during data collection, we had to conduct a number of interviews through telephone, which might have affected the results. Because the absence of face-to-face contact restricted the development of communication, and we couldn’t see body language and facial expressions of interviewees. Thus the resulting data may not be as rich as it would be from a face-to-face interview[49]. Second, interviews in this study were conducted only with women who had vaginal birth.

## Conclusion

According to the results of this study, the birth plan, accompanied with childbirth preparation classes, increases the likelihood of a successful vaginal birth and the desire for having another child. The findings of this study highlight the effectiveness of the birth plan in terms of women’s empowerment for vaginal birth and their satisfaction during childbirth and in the postpartum period. Therefore, vaginal birth promotion policies can include using birth plans in prenatal care of low-risk pregnant women and in the educational content of childbirth preparation classes in Iran. This can be an effective intervention aimed at reducing the rate of cesarean section. Furthermore, it seems that the present study may greatly interest for managers, leaders, and policymakers and may support current population policies in Iran. However, further studies are required to identify barriers to assess the effectiveness of the birth plans.

## Supplementary Information

**Additional file 1**.

## Data Availability

All relevant data are given within the manuscript.

## Abbreviations

COVID-19: Coronavirus disease of 2019
COREQ: Consolidated criteria for reporting qualitative research
